# Understanding the Occurrence and Fate of Atmospheric Microplastics and Their Potential Risks to Human Health: Protocol for a Cross-Sectional Analysis

**DOI:** 10.2196/60289

**Published:** 2024-11-29

**Authors:** Shaikh Sharif Hasan, Abdus Salam, Mohammad Moniruzzaman, Md Aynul Bari, Nirupam Aich, Farjana Jahan, Mahbubur Rahman, Zubayer Islam, Md Humayun Kabir, Md Aftab Ali Shaikh, Rubhana Raqib, Sarker Masud Parvez

**Affiliations:** 1 Health System and Population Studies Division Environmental Health and WASH icddr,b Dhaka Bangladesh; 2 Department of Chemistry University of Dhaka Dhaka Bangladesh; 3 Bangladesh Council of Scientific and Industrial Research (BCSIR) Dhaka Bangladesh; 4 Department of Environmental & Sustainable Engineering University at Albany State University of New York Albany, NY United States; 5 Department of Civil and Environmental Engineering University of Nebraska–Lincoln Lincoln, NE United States; 6 Global Health and Migration Unit Department of Women’s and Children’s Health Uppsala University Uppsala Sweden; 7 Nutrition Research Division icddr,b Dhaka Bangladesh; 8 Children’s Health and Environment Program, Child Health Research Centre The University of Queensland Brisbane Australia

**Keywords:** plastic exposure, microplastic, bisphenol, phthalate, heavy metals, health consequences, potential risk, cross-sectional analysis, pollution, nanoplastics, additives, plastic additives, health consequence, exposure, human exposure, plastic recycling, recycling activities

## Abstract

**Background:**

Plastic pollution has reached an alarming magnitude, defining the contemporary era as the “Plastic Age.” Uncontrolled plastic production and inadequate recycling processes have led to widespread contamination of the environment with micro and nanoplastics.

**Objective:**

The study aims to assess the environmental and human health consequences of exposure to microplastic particles (MPs) and their additives among plastic recycling workers in Dhaka. Specifically, it focuses on mapping the management pathways of plastic waste from collection to disposal, analyzing the types of MPs in the environment, and assessing the potential health impacts on plastic recycling workers.

**Methods:**

A cross-sectional exploratory study design was used, consisting of exposed and nonexposed groups in plastic recycling sites in Dhaka, Bangladesh. The study will establish possible associations between different health consequences and microplastic particle exposure with a systematic approach involving plastic recycling hot spot detection, management pathway mapping, and detecting the presence of environmental MP. MPs and heavy metals will be detected from environmental samples using fluorescence microscopy, Fourier-transform infrared spectroscopy, and inductively coupled plasma mass spectrometry. Human exposure will be assessed by detecting the metabolites of bisphenol and phthalates from urine samples using liquid chromatography–tandem mass spectrometry and thoroughly evaluating endocrine, reproductive, respiratory, and renal functions. The sample size was derived from the mean concentrations of urinary bisphenol and phthalates metabolites, requiring the participation of 168 respondents. A 1:1 exposure to nonexposed stratification would be sufficient to meet our study objectives, considering the conventional level of power and confidence interval. This study protocol (PR#22111) has received approval from the Research Review Committee and Ethical Review Committee of the icddr,b.

**Results:**

The project was funded in August 2022. We started collecting environmental samples in January 2023 and completed participant enrollment, exposure survey, and biological sample collection by December 2023. We enrolled 84 adult plastic recycling workers with at least 5 years of exposure history and 84 nonexposed participants who were not involved with plastic recycling activities. Data analysis is currently underway, and the first results are expected to be submitted for publication in November 2024.

**Conclusions:**

The findings would provide valuable insights into the adverse impacts of microplastic pollution on both the environment and human health, aiding in better understanding the extent of the issue.

**International Registered Report Identifier (IRRID):**

DERR1-10.2196/60289

## Introduction

### Background

Plastic has become essential to our “throwaway” lifestyle. Due to its ubiquitous presence in our environment, the current era is better known as the “Plastic Age” [1]. In 2019, around 368 million metric tons of plastics were produced worldwide, which is expected to reach 33 billion tons by 2050 [2]. Plastic has invaded our natural resources and ecosystems to become a global environmental and public health threat. It is reported that 79% of global plastic products have yet to be appropriately recycled and are eventually released into landfills or natural environments [3]. About 0.8 million tons of plastic waste are generated annually in Bangladesh, among which 36% are recycled, 39% are landfilled, and the rest are considered leakage or unattended [4]. A study reported that 5 to 13 million metric tons of plastic waste was released into the ocean by 192 coastal countries in 2010 [5]. If urgent action isn’t taken, the societal cost of plastic production could hit US $7.1 trillion by 2040, surpassing 2018’s global health spending and the combined 2019 gross domestic product of Germany, Canada, and Australia [6].

Plastic degrades into powdery fragments and microscopic-sized plastics (less than 5 mm) called microplastics [7]. The pollution of the environment with degraded plastics and microplastics is an emerging concern attracting global attention [8]. Exposure to microplastic particles (MP) results in adverse outcomes, which can be classified into physical effects (such as those related to particle size, shape, and concentration) and chemical effects (involving harmful chemicals associated with MPs) [9]. Cox et al reported the presence of an average of 0.44 MPs/g of sugar, 0.11 MPs/g of salt, and 0.09 MPs/g of bottled water in the American diet [10]. Typically, humans consume 80 g of MPs/per day through fruits and vegetables that accumulate MPs through uptake from polluted soil [11]. Among numerous toxic chemicals, bisphenol and phthalates are most commonly used as plasticizers [12], where MPs work as vectors for these chemicals. Plastic and its additives harm human health and the environment [13,14]. Furthermore, many heavy metals like zinc, nickel, mercury, cadmium, and lead are present in plastic compounds as additives and pigments [15]. Long-term exposure to these metals has adverse health consequences [16-18]. In Bangladesh, MP was detected in different fish and shrimp species from the mangrove estuary and the Northern Bay of Bengal [19,20]. An abundant number of MPs were also detected in the beach sediments of Cox’s Bazar, Bangladesh [21]. To our knowledge, limited research exists worldwide on the impacts of atmospheric microplastics, and the study of their human health impacts in Bangladesh is also scarce.

Proper plastic recycling plays a vital role in mitigating the environmental impact of plastic waste and promotes a sustainable future for generations to come. However, the mechanical friction and abrasion of the plastic recycling industries produce tons of MPs [22]. Plastic waste recycling and its harmful effects on human and environmental health should be adequately addressed in Bangladesh. Epidemiological studies indicate that plastic particles and their chemical additives (eg, bisphenol, phthalates, flame retardants, etc) may have adverse impacts on human health [23]. However, the underlying mechanism still needs to be clearly understood. Understanding the extent of environmental exposure to plastic additives and consequential health outcomes would provide valuable knowledge and help to develop effective control measures.

### Objectives

The fundamental objective of this study is to determine the extent of environmental and human health consequences resulting from exposure to plastics (microplastics) and their associated additives. The specific objectives are (1) to identify the factors that determine the location of hot spots with higher exposures and concentrations of MPs in Dhaka city; (2) to map the management pathways of plastic waste in Dhaka city, specifically from collection to disposal; (3) to characterize the nature of plastics that entry into the environmental system of Dhaka city by identifying the types of MPs (composition, size, and shape), their sources, entry points, and contamination levels in different environmental samples; and (4) to evaluate the possible health impacts of MP exposure among workers in the plastic recycling industry.

## Methods

### Study Design and Participant Recruitment

We will use a cross-sectional exploratory study consisting of an exposed and a nonexposed comparison group. This study will be conducted at plastic recycling sites in Dhaka city. The research team will identify major plastic recycling sites. Participants will be recruited according to the following inclusion criteria: (1) workers involved in plastic recycling processes for at least 5 years and (2) aged 18 years or older at the time of data collection. We will exclude irregular part-time workers (eg, those who occasionally work in plastic recycling factories as day labor but primarily rely on other occupations for livelihood) and children under 18 years old. We will also exclude the participants who are on antibiotic treatment as it may mask symptoms of infection and alter the immune response [24,25]. Data collectors will clarify the study objectives, procedures, and intended outcomes to the participants and obtain written consent.

In addition, we will select a nonexposed control site within Dhaka city, where no plastic processing factory exists. Since plastic has become embedded in our daily lives, having a population free from plastic pollution’s effects seems unreasonable. All nonexposed participants will be other occupational workers, including daily laborers, masons, carpenters, etc, who have had no exposure to the plastic industry.

### Sample Size

We calculated the sample size based on the primary outcomes, including bisphenol and phthalate metabolites in biological samples. Mean concentrations of urinary bisphenol and phthalate metabolites from studies with similar outcomes were used [26,27]. Among the phthalate metabolites, urinary mono isononyl phthalate (MiNP) had a mean of 10.12 (SD 4.91) µg/L in plastic recyclers and 12.22 (SD 4.69) µg/L in the general population. The estimated sample size was 168 (84 per group) using the 2-sample mean test, considering 80% power and 95% CI.

### Data Collection Methods

The detailed study activities by study objectives are discussed below.

#### Objective 1: Hot Spot Identification

Hot spot identification is the initial and crucial step in plastic waste management. The field team has identified the plastic recycling facilities across Dhaka and recorded the GPS coordinates. This data was compiled and analyzed using ArcGIS Pro mapping software (version 3.3; Environmental Systems Research Institute). The research team also sought permission from the plastic recycling factory owners to conduct the research within their facilities.

#### Objective 2: Mapping the Management Pathway

Plastics are typically distributed into three categories: (1) plastics in active use, (2) postconsumer managed plastic waste, and (3) mismanaged plastic waste [28]. Usually, plastic waste that is managed undergoes disposal methods such as recycling, incineration, or landfilling. However, improperly managed plastic waste is either discarded into the environment or inadequately disposed of and often ends up in rivers and oceans, contributing adversely to the ecosystem [29]. To identify the current challenges within Dhaka city’s plastic waste management system, the research team will interview key informants involved in collection, refurbishment, reuse, crude recycling, and disposal. The team will collect information on who collects plastics, the primary source of collection (such as homes, institutions, dump sites, and transfer stations), the types of plastics collected, transportation methods to the recycling center, which part of plastics are reused and recycled, and the ultimate disposal site and methods of disposal (burying, incineration, or chemical melting). Purposive sampling techniques will be used, and data will be collected using 2 tools: in-depth interviews and key informant interviews. Sampling will continue until information redundancy or saturation is attained [30].

#### Objective 3: Quantifying the Extent of Environmental Pollution

The research team will collect different environmental samples, including floor dust and topsoil (0- to 5-cm depth) from the plastic recycling and dismantling sites, sediments from the water vicinity (lake, river, or pond) where such facilities are operated, and air samples (Table 1). The detailed sampling procedures are described below.

**Table 1:** Human participants and environmental sampling plan.

Environmental samples		Proposed number
**Exposed group (proposed: n=84)**		
	Air	186
**Nonexposed group (proposed: n=84)**		
	Soil	20
	Dust	20
	Sediment	10

##### Air Sampling

The field research team conducted air sampling at 2 plastic recycling industries or shops and 1 outdoor location for MPs and heavy metals (HMs) detection. Air sampling was performed using a filter-based low-volume PM_2.5_ (particulate matter with a diameter less than 2.5 µm) sampler for heavy metal detection in nylon 6.6 filter paper, and a total suspended particulate (TSP) sampler was used for microplastic detection in the quartz fiber filter. The team set up PM_2.5_ and TSP samplers in each selected industry or shop and outdoor location with a duration of 6 hours and a frequency of once every 6 days to assess the work-related exposure of factory workers. We collected air samples in both dry (January to March) and wet seasons (June to August) to explore seasonal variation. After sampling, quartz fiber filters were kept in glass filter holders and nylon filters in Ziplock bags. All the samples were placed in a cool box and kept at 4 °C till transported to the laboratory. The samples were preserved at a negative 20 °C temperature at the laboratory for future analysis.

##### Soil Sampling

We will develop a 10×10 square meter grid map of selected plastics recycling factories to collect soil samples. The team will collect 20 soil samples from the selected areas of the grid map. We will identify the closest open-air bare soil surface using a whisk broom (nonplastic) and clean about 1 m^2^ of area. Using the stainless-steel hand trowel, the clean surface will be divided into 6 equal quadrangles. A total of 6 subsamples will be collected from these quadrangles at depths ranging from 0-5 cm depth using a stainless-steel auger and placed in a metal tray. After thoroughly mixing the subsamples and removing stones and other nonsoil materials, approximately 100 g of soil sample will be enclosed in aluminum foil, put in a Ziploc bag, and labeled appropriately. Samples will be kept in a cooler box until they are carried to the laboratory, where they will be preserved at a negative 20 °C for later analysis.

##### Dust Sampling

The field team will collect indoor dust samples from the plastic recycling shop or industry where we have recruited our study participants. Our field research team will approach the shop or industry at the end of working hours but before cleaning to collect dust samples. We will sweep the floor under desks, shelves, and other areas, including furniture, machinery, and walls, collecting dust using a dustpan with a precleaned nonplastic brush. A 100-g sample will be obtained from the dust that remains after the sample has been mixed using a metallic hand trowel. These samples will be enclosed in aluminum foil, put in a Ziploc bag, and labeled appropriately. We will keep the samples in a cooler box until they are carried to the laboratory and preserve them at negative 20 °C for later analysis.

##### Sediment Sampling

The team will collect sediment samples from the nearest water body in the selected plastic recycling industries. Following the same 10×10-meter grid map, the field team will identify 10 sampling locations. Surface sediments will be collected at 0- to 20-cm depth using a metallic handheld sediment sampler. Samples will be collected in a glass jar labeled appropriately, put in a Ziplock bag, and transported to the laboratory for later analysis.

##### Environmental Sample Analysis

For microplastic detection from environmental samples, an adequate amount of samples will be combined with about 20 mL of 35% H_2_O_2_ and heated at 60 °C for an hour for organic matter removal. For density separation, 20 mL NaCl solution will be added, ultrasonicated for 20 minutes, and left overnight for organic matter removal. The top solution will be filtered through quartz and examined under an Oxion Inverso microscope (40× to 100×; Euromex Microscopen BV). The filter, post examination, will be stained using Nile red solution (1-5 mg/mL) and observed under green excitation fluorescence (540-580 nm). Microplastic sizing will be conducted using the ImageJ software (LOCI, University of Wisconsin).

Environmental samples will be analyzed for heavy metal detection using inductively coupled plasma mass spectrometry (ICP-MS) following the acid digestion method. Samples will be dried at 105 °C until reaching a stable weight, followed by disaggregation using a mortar and pestle and then sieving. Following the US Environmental Protection Agency (EPA) Method 3051, fine fraction samples weighing 0.3 grams will be subjected to acid digestion using a microwave oven digester. The resulting digested samples will be analyzed using an ICP-MS (NexION 2000, PerkinElmer).

#### Objective 4: Health Risk Characterization

Plastic particles enter the human body through inhalation, ingestion, and dermal contact [31]. In the human body, they cause oxidative stress [32,33], cytotoxicity [34,35], and even translocation to different tissues [36]. There is limited evidence on the direct human health effects of MP exposure, but mathematical modeling, animal studies, and in vitro cell cultures revealed potential health risks like altered metabolism, immune disruption, neurotoxicity, and reproductive toxicity [37]. Previous biomonitoring approaches have demonstrated the presence of bisphenol, phthalates, and other plastic additives and their metabolites in the human population. This study focuses on 2 common types of plastic additives: phthalates and bisphenol metabolites and various heavy metals. Based on the literature review, the human health systems most affected are identified and listed in Table 2. To assess the impact on human health, we will collect biological samples, conduct anthropometric measurements, measure blood pressure, and assess the lung function of study participants.

**Table 2:** Major systems affected and the potential health impact assessment plan following exposure to microplastics and their additives.

Health systems and health-related outcome		Disease indicators	Biological sample	Procedure
**Female reproduction**				
	Hormonal imbalance	↑ Progesterone, ↑ LH^a^	Fasting blood	ELISA^b^
	Undesirable pregnancy outcomes	↑ Risk of recurrent miscarriage, ↑ Spontaneous preterm birth, ↑ Preterm premature rupture of membrane	—^c^	Self-reported questionnaire
**Male reproduction**				
	Hormonal imbalance	↓ Serum total testosterone ↑ Prolactin	Fasting blood	ELISA
	Sexual dysfunction and infertility	↓ sexual function, ↓ sexual desire, ↓ erectile ability, ↑ premature ejaculation	—	Self-reported questionnaire
**Endocrine**				
	Metabolic disorder (any 3 from the 5 features are suggestive of metabolic disorder)	Height, weight, waist, and hip circumference	—	Anthropometry
	Metabolic disorder (any 3 from the 5 features are suggestive of metabolic disorder)	Triglycerides	Fasting blood	Semiautomatic biochemistry analyzer
	Metabolic disorder (any 3 from the 5 features are suggestive of metabolic disorder)	HDL^d^ cholesterol	Fasting blood	Semiautomatic biochemistry analyzer
	Metabolic disorder (any 3 from the 5 features are suggestive of metabolic disorder)	Blood pressure	—	Automated blood pressure monitor
	Metabolic disorder (any 3 from the 5 features are suggestive of metabolic disorder)	Fasting blood glucose	Fasting blood	Hexokinase method
	Thyroid function	↓T4^e^, ↓TSH^f^	Fasting blood	ELISA
**Respiratory**				
	Asthma	↓ Lung function	—	Spirometry
**Renal**				
	Impaired renal function	↑ Albuminuria	Fasting urine	Dipstick test for albumin
	Impaired renal function	Urinary creatinine	Fasting urine	Automated hematology analyzer
	Impaired renal function	Urinary micro-albumin	Fasting urine	Automated hematology analyzer
	Impaired renal function	↓ eGFR^g^	Blood	CKD-EPI^h^ equation
**Oxidative stress**				
	DNA damage	8-OHdG^i^	Fasting urine	ELISA

^a^LH: luteinizing hormone.

^b^ELISA: enzyme-linked immunosorbent assay.

^c^Not available.

^d^HDL: high-density lipoprotein.

^e^T4: thyroxine.

^f^TSH: thyroid stimulating hormone.

^g^eGFR: Estimated Glomerular filtration rate.

^h^CKD-EPI: Chronic Kidney Disease Epidemiology Collaboration.

^i^8-OHdG: 8-hydroxy-2-deoxyguanosine.

#### Biological Sample Collection, Assessment, and Survey

Upon informed consent, a trained team collected 50 mL of fasting urine samples in urine collection pots to detect bisphenol and phthalate metabolites [38]. Total bisphenol (free and conjugated) and phthalate metabolites will be determined by liquid chromatography–tandem mass spectrometry (LC-MS/MS) using the Agilent 6420 Triple Quad LC/MS System. The trained field team also collected 5 mL fasting venous blood samples using trace metal-free vacutainers, and an additional 3 mL was collected using a serum tube. All the samples were carried in a cooler box (4-8 °C) during transportation and stored in a negative 80 °C freezer until analysis.

We also developed a data collection tool and piloted it before applying it in the field. Our research team administered electronic questionnaires, gathering data across multiple domains. This includes general characteristics such as age, gender, income, education, and living environment, as well as detailed exposure histories and attitudes toward plastic waste. We also recorded lifestyle factors like smoking and medication, along with self-reported health conditions such as hypertension, diabetes, and liver diseases. Interviews were conducted at the participants’ workplaces.

### Blood Pressure Measurement

The team used an automated blood pressure monitor to assess the blood pressure of all participants, ensuring that all necessary precautions were taken care of. Participants were instructed to refrain from consuming caffeine (such as tea, coffee, and carbonated beverages), engaging in heavy physical activities, and smoking for at least 30 minutes before measuring blood pressure. They were then asked to rest for 5 minutes while seated on a chair with arm support. Different cuff sizes and calibrated instruments were used according to participants’ age groups. Blood pressure measurements were conducted thrice, initially from the left arm, followed by the right arm, and then once more from the left arm. Systolic blood pressure and diastolic blood pressure readings were noted separately, and then the readings were averaged.

### Respiratory Measurement

The research team collected information on smoking habits and medical history, including any previous diseases. The questionnaire inquired about acute symptoms, such as eye irritation, nasal congestion, sore throat, and headache. Lung function was assessed using a portable digital spirometer, adhering to standard operating procedures. Measurements encompassed forced vital capacity (FVC), forced expiratory volume in one second (FEV_1_), and peak expiratory flow rate (PEFR).

### Dietary Diversity Assessment

Previous research findings indicate that humans are commonly exposed to microplastics through inhalation, ingestion, and skin contact [23]. Thousands of chemicals and heavy metals are added to plastics as plasticizers; they enter the human body directly with microplastics. It was estimated that an American consumes an average of 39,000 to 52,000 MPs annually, where water was found to be the most prominent source [10]. Even our regular food items like chicken meat and fish were found to be contaminated with microplastics [39]. So, exploring environmental pollution and health consequences will be incomplete without a dietary assessment. Dietary diversity was measured considering the 1-week recall period. This assessment will guide us to the probable sources and help us measure the risks more precisely.

#### Statistical Methods and Data Analysis

Data analysis will be performed using the “R” programming language (version 3.5.2; R Foundation for Statistical Computing) and Stata (version 14; StataCorp). A codebook will be prepared based on data collection questionnaires. All variable labels, value labels, and composite variable calculations will be constructed using do files and “R” scripts. Throughout the data collection phase, quality control measures will be implemented. Any anomalies will be promptly addressed through discussion and resolution. Data will be summarized using descriptive statistical tools such as mean (SD)for quantitative symmetric variables, median (IQR) for quantitative asymmetric variables, and frequencies or proportions for qualitative variables. The chi-square test will be used to assess the association between 2 categorical variables [40]; independent *t* tests will be used to determine the significance of the differences between the means of 2 groups for normally distributed data [41], whereas the Kruskal-Wallis rank test will be used to compare the median between 2 groups or variables for non-normally distributed data [42]. After adjusting for the relevant covariates, inferential analyses such as regression and risk ratio will be calculated to draw more definitive conclusions. Confounding variables will be identified from the literature, and different types of bivariate analysis will be included in the regression model.

For qualitative data analysis, we will use ATLAS.ti (version 5.2; ATLAS.ti Scientific Software Development) to organize our data and conduct thematic analysis [43]. Our approach will begin with transcription and familiarization with interview content, followed by selecting significant quotations and identifying keywords. We will develop a coding system by iteratively reading and coding the transcripts to uncover patterns and themes. These coded segments will be entered into ATLAS.ti to facilitate theme identification and further analysis. Themes will be developed by grouping related codes and interpreting their meaning in context, aiming to construct a conceptual model that illustrates connections and insights from the data. Throughout this process, we will compare themes across interviews to ensure comprehensive coverage and triangulate findings for enhanced validity and reliability [44].

#### Ethical Considerations

The Research Review Committee (RRC) and Ethical Review Committee (ERC) of the icddr,b have reviewed and approved this study protocol (PR#22111). Eligible participants who consent to participate will be asked to provide written consent in the local language (Bengali). There will be no compensation for their participation in the research. Interviews will be conducted in private or in a comfortable setting to minimize the risk of confidentiality breaches. The interviewers will use culturally appropriate terminology and make every effort to ensure the subjects’ comfort. Participants will be informed of their option to discontinue their participation in the study at any time if they feel uncomfortable. To protect participants’ privacy, anonymous data sets without personal identifiers will be developed, and deidentified data will be used during storage, analysis, and dissemination.

## Results

### Overview

This project was funded in August 2022. Following approval from the RRC and ERC, the data collectors were recruited, and a comprehensive exposure survey and sample collection training was provided. Before starting fieldwork, we developed and piloted the data collection tool. We initiated air sample collection in January 2023 and completed this phase by August 2023. Simultaneously, biological sample collection and exposure surveys were started in June 2023 and completed by October 2023. Additional environmental samples, including soil, dust, and sediment from the exposure sites, were collected by May 2024. Data analysis is ongoing, and we anticipate publishing our first result by November 2024.

We successfully enrolled a total of 84 exposed and 84 nonexposed respondents, with sociodemographic information detailed in Table 3. The mean age of exposed respondents was 34 years (SD 6.47), slightly younger than the nonexposed respondents (mean 36, SD 13.3 years) population. The majority of the respondents were male, and almost half of them did not have any formal education across exposed and nonexposed groups. The BMI of nonexposed respondents was slightly higher than exposed respondents; however, this difference was not significant (*P*≥.05).

Currently, all biological and environmental samples are undergoing analysis in the laboratory, and we anticipate obtaining the results by July 2024.

**Table 3:** Participants characteristics of exposed and nonexposed population.

Characteristics		Exposed population (N=84), n (%)	Nonexposed population (N=84), n (%)	*P* value
Age in years, mean (SD)		34.3 (6.47)	35.7 (13.3)	.9^a^
Sex, male, n (%)		49 (58.3)	49 (58.3)	.01^b^
**Education, n (%)**				
	No education	53 (63.1)	47 (56)	.19^b^
	Up to primary school	28 (33.3)	28 (33.3)	.19
	Above primary school	3 (3.6)	9 (10.7)	.19
	Married, n (%)	72 (85.7)	71 (84.5)	.31^b^
	Self-owned home, n (%)	29 (34.5)	32 (38.1)	.52^b^
	Family members, mean (SD)	4.31 (1.92)	4.55 (1.70)	.39^a^
	Monthly income (US $), median (IQR)	182 (136-255)	182 (168-273)	.05^c^
**Households have their own, n (%)**				
	Television	0 (0)	1 (1.2)	.32^b^
	Mobile phone	27 (32.1)	37 (44)	.11^b^
	Refrigerator	37 (44)	46 (54.8)	.17^b^
	Electric fan	82 (97.6)	83 (98.8)	.56^b^
	Current smoker, n (%)	31 (36.90)	22 (26.19)	.14^b^
	Alcohol consumption, n (%)	10 (11.9)	3 (3.57)	.04^b^
	BMI (kg/m^2^), median (IQR)	22.9 (19.5-25.6)	23.6 (21.4-26.4)	.66^c^

^a^Independent sample *t* test.

^b^Chi-square test.

^c^Mann-Whitney *U* test.

### Dissemination

The study results will be disseminated to the Director General of Health and Family Services, the Department of Environment (DoE), and partnering civil society organizations in Bangladesh that are dedicated to addressing health and environmental issues, aiming to uphold environmental safety. Findings will be used to explore the boundaries and challenges of plastic recycling and management in Bangladesh. Furthermore, strategies at both technical and policy levels will be formulated alongside efforts to enhance public awareness of the environmental and health risks linked with plastic recycling. We will also develop initiatives to build capacity in this area. Abstracts will be prepared for international conferences to disseminate our findings globally, and manuscripts will be drafted and submitted to peer-reviewed journals for publication.

## Discussion

### Principal Findings

Several studies have examined the issue of plastic pollution in Bangladesh, with a primary focus on marine and aquatic environments. However, no study has investigated the potential human health impacts resulting from microplastic exposure [45]. Earlier evidence indicates the presence of MP in fish [20] and shrimp [46] from the northern Bay of Bengal. In addition, microplastics have been detected in sediments from Bangladesh’s sea beaches and major rivers [47]. While exploring the sources of these microplastics, it was revealed that the plastic recycling industry also contributes due to the mechanical process and inadequate effluent treatments [48].

The presence of microplastic particles in our surrounding environment is evident, yet numerous unexplored aspects remain in measuring exposure and assessing health outcomes. While most of the health impacts have been hypothesized based on animal studies, only a few have been investigated through epidemiological research in humans. The proposed study aims to contribute evidence on human microplastic exposure and possible health impacts suggested by animal studies. In 2022, researchers from the Netherlands reported the first-ever detection of plastic particles in human blood, with 77% of the tested blood samples containing such particles [49]. Instead of directly detecting microplastic from biological samples, this study will focus on the metabolites of bisphenol and phthalates [50]. Furthermore, this study is designed to generate evidence on the presence of microplastic particles in environmental media, the extent of human exposure, and associated health outcomes. Collecting and analyzing a wide range of environmental sample types will contribute to a more comprehensive understanding of microplastic distribution in the environment and potential routes of human exposure. The results from this study will serve as a foundation for further research into monitoring the long-term ecological impacts of plastic debris. The multifaceted approach will also enhance the ability to develop targeted strategies for future mitigation and regulatory measures.

Nonetheless, this study has some limitations. We intend to identify the presence of bisphenol and phthalate metabolites in biological samples, representing an indirect method of assessing exposure. To address this limitation, we plan to collect environmental samples from the respondents’ workplaces to detect the presence of MPs, which may validate our exposure assessment. This study is cross-sectional, from which causality between exposures and outcomes cannot be inferred.

### Conclusion

The findings from this study could be valuable for regulatory authorities in understanding the magnitude of the problem. The proposed analysis of multiple systems, including hormonal, renal, lung function, and oxidative stress indicators, may enhance our understanding of the mechanism between exposures and biological responses. In addition, the detailed environmental investigation would provide initial evidence of contamination in the plastic industry, which might lead to negative consequences for the environment and human health. These findings could also be crucial in implementing occupational safety measures to mitigate adverse impacts, enabling informed decisions in formulating effective waste management and resource use strategies.

## Appendix

Multimedia Appendix 1 Peer review report by the Research Review Committee (RRC) - International Centre for Diarrhoeal Disease Research, Bangladesh (icddr,b).

## Abbreviations

DoE: Department of Environment
EPA: Environmental Protection Agency
ERC: Ethical Review Committee
FEV1: forced expiratory volume in one second
FTIR: Fourier-transform infrared spectroscopy
FVC: forced vital capacity
HM: heavy metal
ICP-MS: inductively coupled plasma mass spectrometry
LC-MS/MS: liquid chromatography–tandem mass spectrometry
MiNP: mono isononyl phthalate
MP: microplastic particles
PEFR: peak expiratory flow rate
PM2.5: particulate matter with a diameter less than 2.5 µm
RRC: Research Review Committee
TSP: total suspended particulate

## Data Availability

The data sets generated and analyzed during this study are available from the corresponding author upon reasonable request.
