# Supplementary material for: Understanding the Occurrence and Fate of Atmospheric Microplastics and Their Potential Risks to Human Health: Protocol for a Cross-Sectional Analysis
Source: JMIR Res Protoc. 2024 Nov 29;13:e60289. doi: 10.2196/60289 (PMC11645502; doi:10.2196/60289)
Supplement: Multimedia Appendix 1 [file resprot_v13i1e60289_app1.pdf]

icddr,b

RESEARCH REVIEW COMMITTEE
Comments Letter

Memorandum

06 October 2022

To: Mr Sarker Masud Parvez
Principal Investigator of research protocol # PR-22111
Infectious Diseases Division (IDD)

From: Shafiqul Alam Sarker, MD, PhD, FRCP
Chairperson
Research Review Committee (RRC)

Sub: **Research protocol # PR-22111**

Thank you for submitting your research protocol # PR-22111 titled "Understanding occurrence and fate of atmospheric microplastics and their potential risks to human health" for consideration by the RRC and present it before the Committee in its special RRC meeting held on 22 September 2022. This is to inform you that after review and discussion, the committee made the following observations on the protocol:

a) "Potential risks on human health" is very generic and the cross-sectional study proposed by the investigators is unlikely to assess numerous health consequences that microplastics (from plastic pollution) may induce. Please specify the health risks (outcomes) that this study is exploring. Are the researchers exploring "environmental health"? if so, please specify what outcome indicators are used for environmental health.

b) In investigator will select participants from plastic processing factories (exposed group) from the exposed site. But it is not clear who will be the non-exposed participants included in the study. How would the non-exposed control site be selected? Who would be selected from the non-exposed site? How would the researchers ensure or objectively assess that the participants selected from the control site truly do not have exposure to atmospheric microplastic before comparing the health outcomes between the two groups? The design and sampling process should be revisited.

c) By interviewing the key informants involved in plastic management the investigators would be able to establish the pathway of plastic that is disposed of or recycled. But it is not clear if and how they would identify the pathway of leakage or unattended plastics.

d) How would they distinguish that the toxic trace metals are from plastic but not from other sources?

e) How would the investigators adjust for potential confounders to the exploratory exposure-outcome relationship in this cross-sectional study? Is there any *a priori* list of confounders? What are they? How will the investigators collect information on those confounders?

f) Although this is an exploratory study, what were the assumptions for selecting 100 vs 50 exposed and non-exposed participants to assess the association with health outcomes? Would this number be enough to identify any indicative relationship adjusting for confounding?

You are, therefore, advised to address each of the above-mentioned observations of the committee and submit the revised version of the protocol for consideration by the chair.

Thank you once again

Cc: Senior Director (Acting), IDD
